# The Spectrum of Neurological Manifestations of Human Herpesvirus 6 Infection in Children

**DOI:** 10.7759/cureus.17183

**Published:** 2021-08-14

**Authors:** Nidheesh Chencheri, Mohammed Dirawi, Saja Tahir, Jwan Shekhy, Walid Abuhammour

**Affiliations:** 1 Department of Pediatric Neurology, Al Jalila Children's Specialty Hospital, Dubai, ARE; 2 Department of Pediatrics, Al Jalila Children's Specialty Hospital, Dubai, ARE; 3 Department of Pediatrics, Al Jalila Children’s Specialty Hospital, Dubai, ARE; 4 Department of Infectious Diseases, Al Jalila Children's Specialty Hospital, Dubai, ARE

**Keywords:** human herpesvirus 6, encephalitis, ganciclovir, roseola infantum, acute disseminated encephalomyelitis

## Abstract

Human herpesvirus 6 (HHV-6) is a member of the *Herpesviridae *family. There are two HHV-6 species: HHV-6A and HHV-6B. HHV-6B causes the majority of documented primary infections and reactivation events. In this case series, we illustrate the varied spectrum of clinical and radiological features of HHV-6 encephalitis and its management in children.

We have described three cases of HHV-6 encephalitis in the age group between nine months and two years. All had an HHV-6 viral load detected in cerebrospinal fluid (CSF) samples. Two of which are of immunocompetent patients.

This case series highlights the importance of including HHV-6 infection as one of the differential diagnoses in a child with suspected central nervous system infection and of considering adding CSF HHV-6 polymerase chain reaction (PCR) test for detection. Increasing awareness of this condition will aid physicians in the timely diagnosis and early treatment of HHV-6 encephalitis.

## Introduction

Human herpesvirus 6 (HHV-6) is a lymphotropic virus, a member of the *Herpesviridae* family. There are two HHV-6 species: HHV-6A and HHV-6B. HHV-6B is a common cause of febrile illness in children worldwide. The classic manifestations of primary HHV-6 infection in immunocompetent children are roseola infantum (sixth disease) and acute febrile illnesses with or without a rash [1]. HHV-6 infects most children within the first two years of life [2-3]. 

Although HHV-6 infection usually causes a benign febrile illness; however, it might cause various diseases of the central nervous system [3]. HHV-6-related encephalitis has been commonly described as a disease solely manifesting in immunocompromised patients. Rarely, it occurs as a complication of roseola or primary manifestation of HHV-6 infection in immunocompetent hosts [4-5]. Misdiagnoses of HHV-6 encephalitis could be attributed to failure to conduct cerebrospinal fluid (CSF) HHV-6 polymerase chain reaction (PCR) test in all suspected CNS-infected children.

The evidence associating HHV-6 with cases of encephalitis of unknown cause has grown increasingly persuasive. With multiple case reports involving adult patients [6], there are only a few case reports of HHV-6 encephalitis in immunocompetent children. We are adding these three cases to this list of reported cases.

## Case presentation

Case 1

A nine-month-old male child presented to our hospital with a history of one episode of generalized tonic-clonic seizure, which lasted for two minutes. He had a background history of high-grade fever and cough for the past seven days. The child was sleepy with poor oral intake for the last two days. On examination, he was febrile and lethargic, but his vitals were otherwise stable. There was no rash or pallor. The cranial nerves examination was unremarkable. He had a low tone with well elicitable deep tendon reflexes. Other systems' examinations were unremarkable. 

The infectious workup showed leukocytosis with a total leucocyte count of 14.8 x 10^9^/L (normal range: 4.0-11.0 x 10^9^/L) and elevated C-reactive protein of 12 mg/L (normal range: 0-5 mg/L). The CSF analysis showed normal CSF glucose (82 mg/dL) and protein (16 mg/dL), and cell count was 2 (all lymphocytes). However, the CSF polymerase chain reaction (PCR) was positive for human herpesvirus 6 (HHV-6). Blood, urine, and CSF cultures were negative. HHV-6 antibodies in blood were negative. Magnetic resonance imaging (MRI) showed mild leptomeningeal contrast enhancement, keeping with the clinical diagnosis of viral meningoencephalitis.

The patient was initially started on IV acyclovir and ceftriaxone as empirical treatment of meningoencephalitis. When cerebrospinal fluid PCR was positive for HHV-6, ganciclovir was initiated. However, his course of treatment withganciclovir was complicated with anemia and severe neutropenia. Therefore, ganciclovir was withheld for one day, and one dose of granulocyte colony-stimulating factor (GCSF) was administered. After completion of 14 days of ganciclovir, the patient showed marked clinical improvement. He was active, alert, and feeding well. The CSF analysis was repeated, and HHV-6 was not detected. Hence, the patient was discharged.

Case 2

A two-year-old male child presented with a one-day history of unsteady gait, upper limb tremors, lethargy, and persistent vomiting. At presentation, there was no history of seizure, photophobia, vision changes, or skin rash. The child appeared lethargic with left-sided torticollis. In addition, he exhibited an ataxic gait, with a tendency to fall on the left side. His neurological examination discovered significant exaggerated deep tendon reflexes and bilaterally sustained ankle clonus. Complete blood count showed normal values. CSF analysis showed positive PCR for human herpesvirus 6 (HHV-6), glucose level was 90 mg/dL (serum glucose level was 171 mg/dL), protein level was 16 mg/dL, white blood cells of 28/uL, polymorphic cells of 4%, and lymphocytes of 96%. Both blood and CSF cultures and HHV-6 serum antibodies were negative. In view of his presentation, a workup for immune deficiencies had been conducted, which was unremarkable.

**Figure 1:**
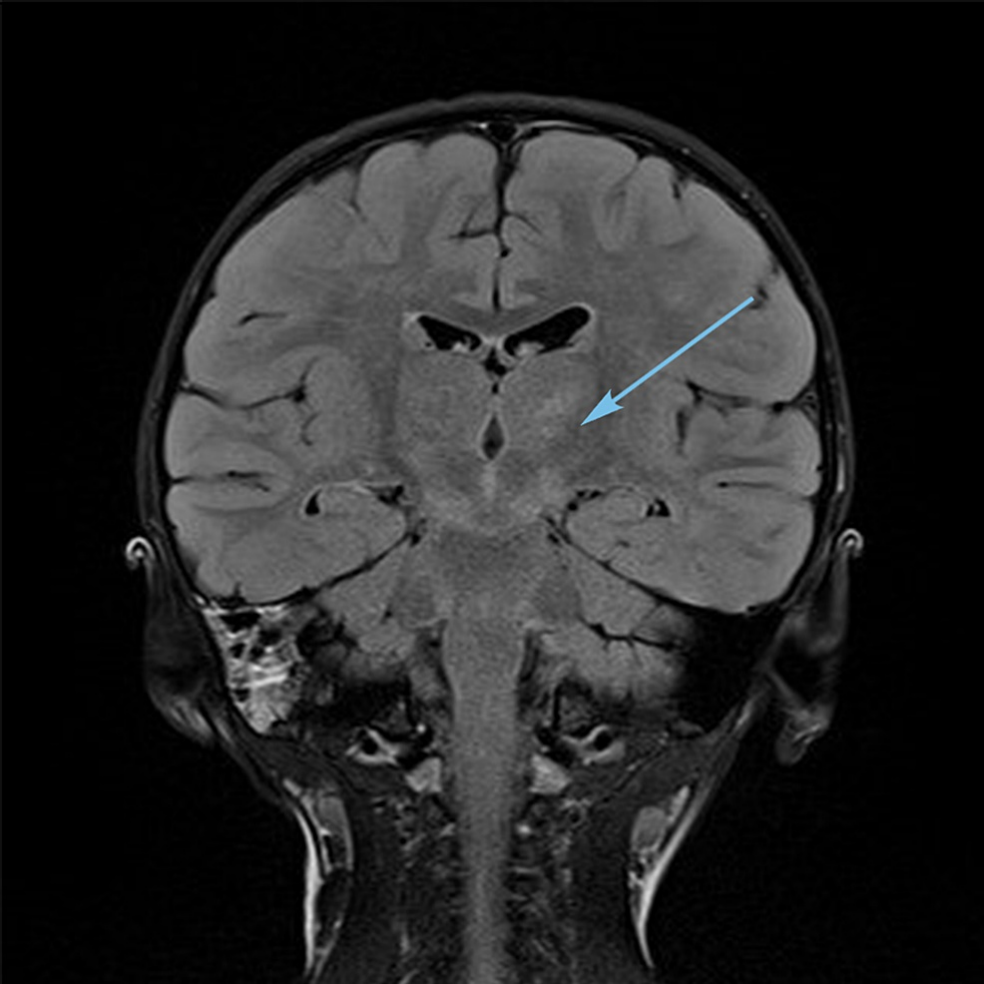
MRI brain T2 coronal FLAIR image showing thalamic and subthalamic hyperintensities (arrow). FLAIR, fluid-attenuated inversion recovery.

**Figure 2:**
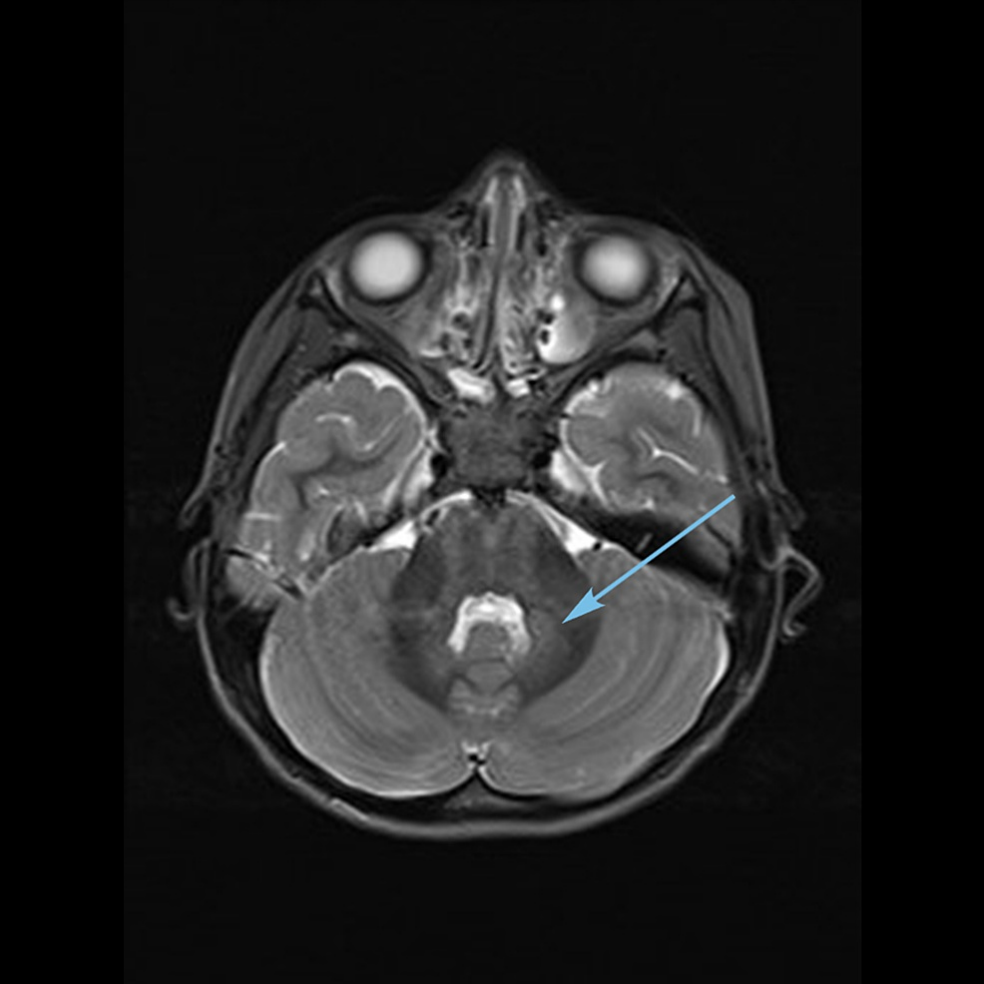
MRI brain T2 axial image showing dentate nuclei hyperintensities (arrow).

**Figure 3:**
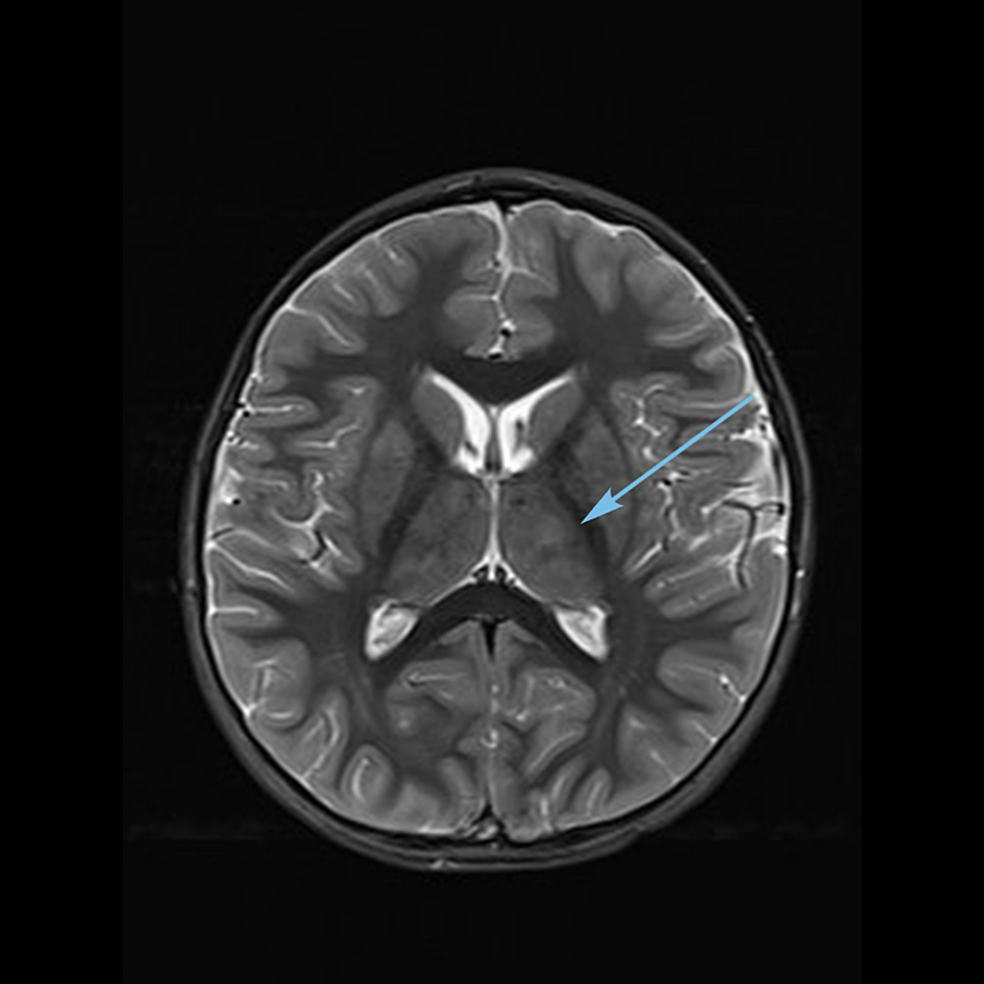
MRI brain T2 axial image showing thalamic hyperintensities (arrow).

Magnetic resonance imaging (MRI) (Figures 1-3) showed multiple bilateral T2 hyperintense lesions over the deep white matter, basal ganglia, thalami, hypothalamus, and subthalamic regions. Subtle involvement of the cerebral peduncles, cerebellar white matter, and dorsal parts of the brain stem was also noted. There was no cortical involvement. The involved areas did not show contrast enhancement.

Intravenous ganciclovir and intravenous immunoglobulin (IVIG) (1 gram/kg daily for two days) were administered. The patient showed marked clinical improvement following treatment with IVIG, 10 days course of ganciclovir, and 10 days valganciclovir upon discharge. A repeat* *neuroimaging* *showed interval improvement in the disease process.

Case 3

**Figure 4:**
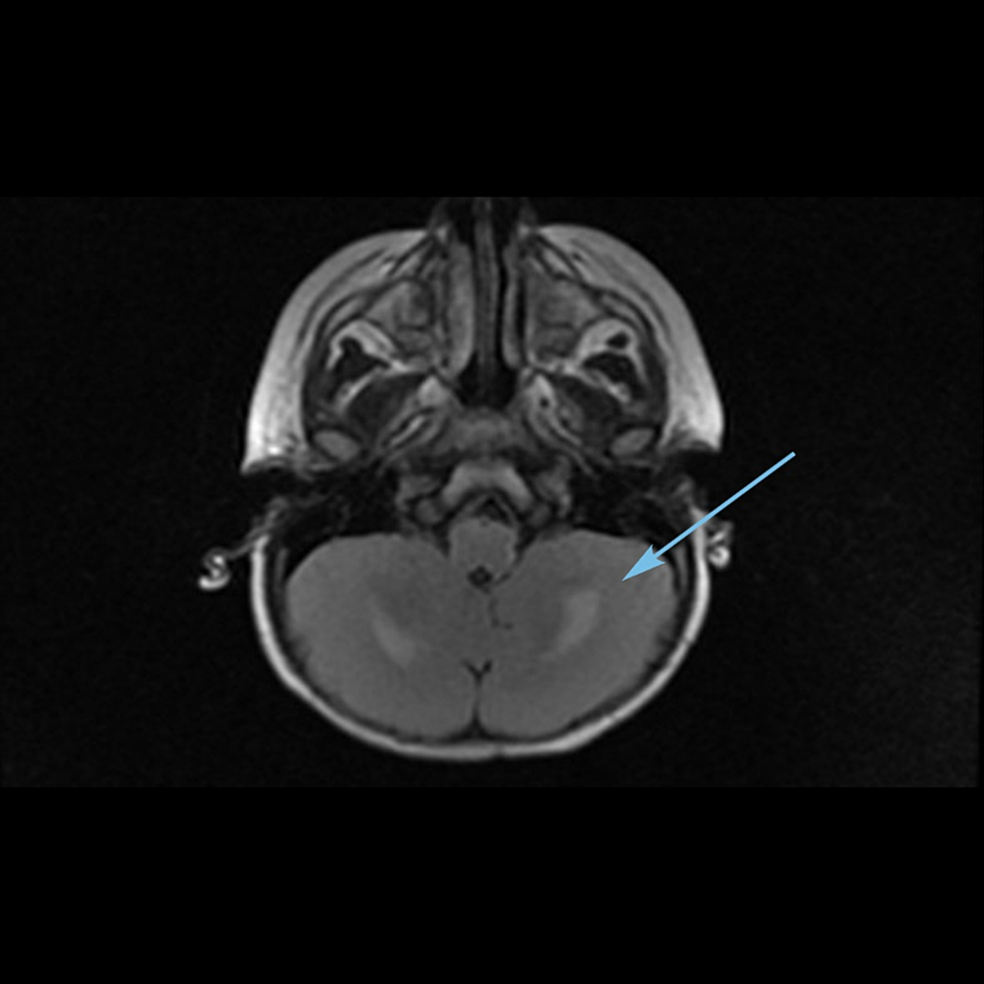
MRI brain axial T2-weighted image showing hyperintensities in the dentate nuclei (arrow).

**Figure 5:**
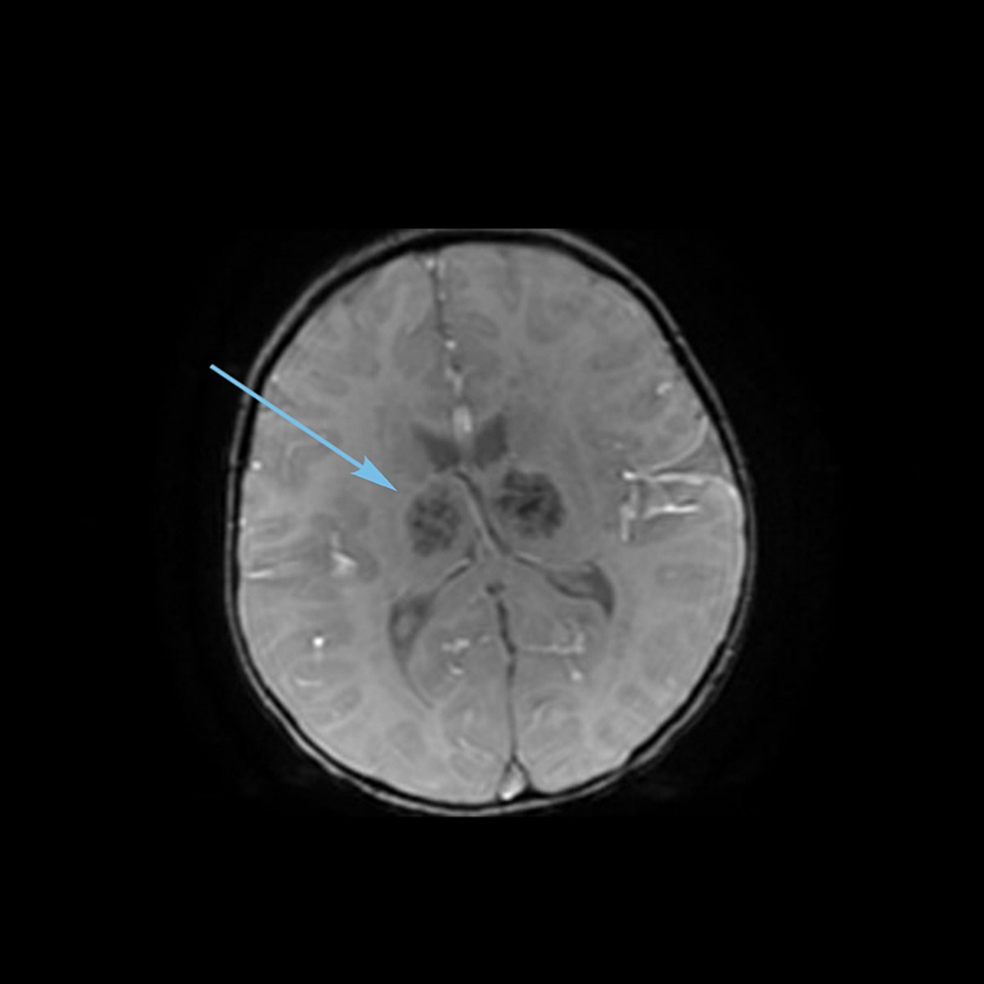
MRI brain axial T2-weighted image showing hyperintensities and edema in bilateral thalami (arrow).

**Figure 6:**
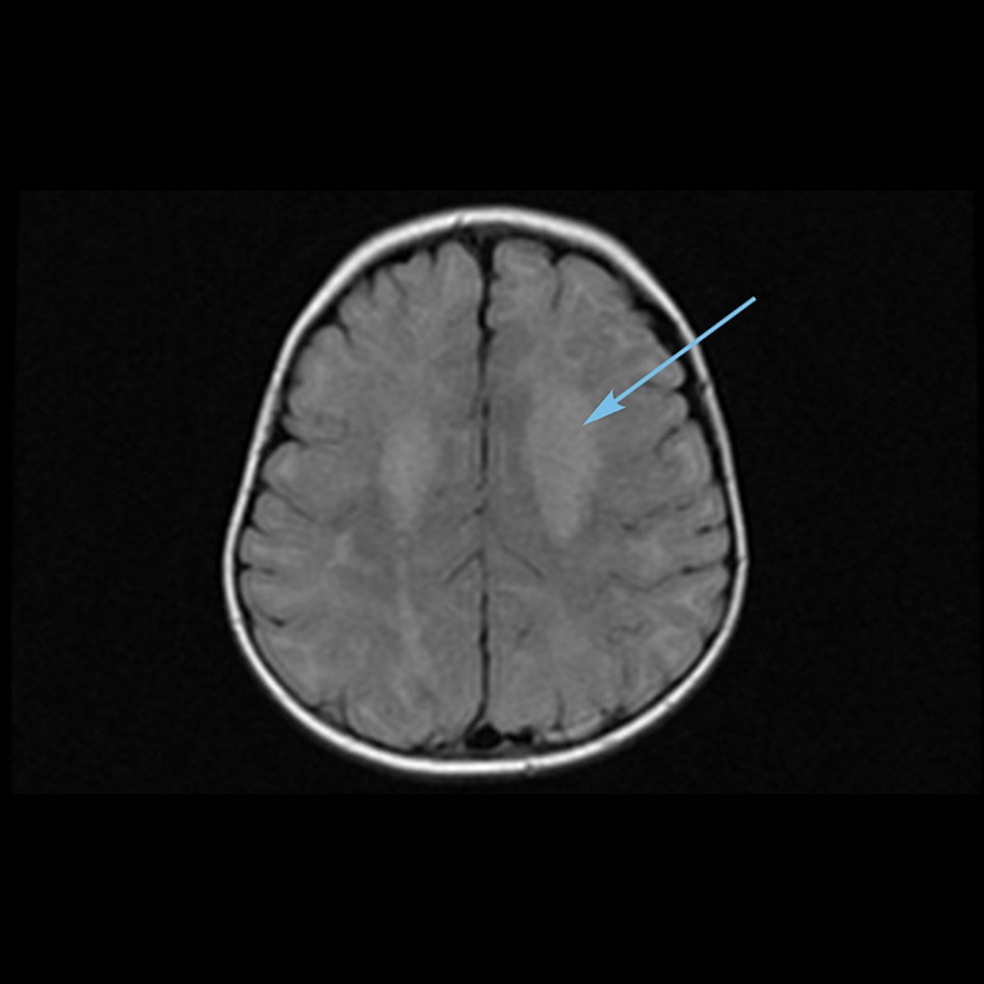
MRI brain axial T2-weighted image showing hyperintensities in centrum semiovale (arrow).

**Figure 7:**
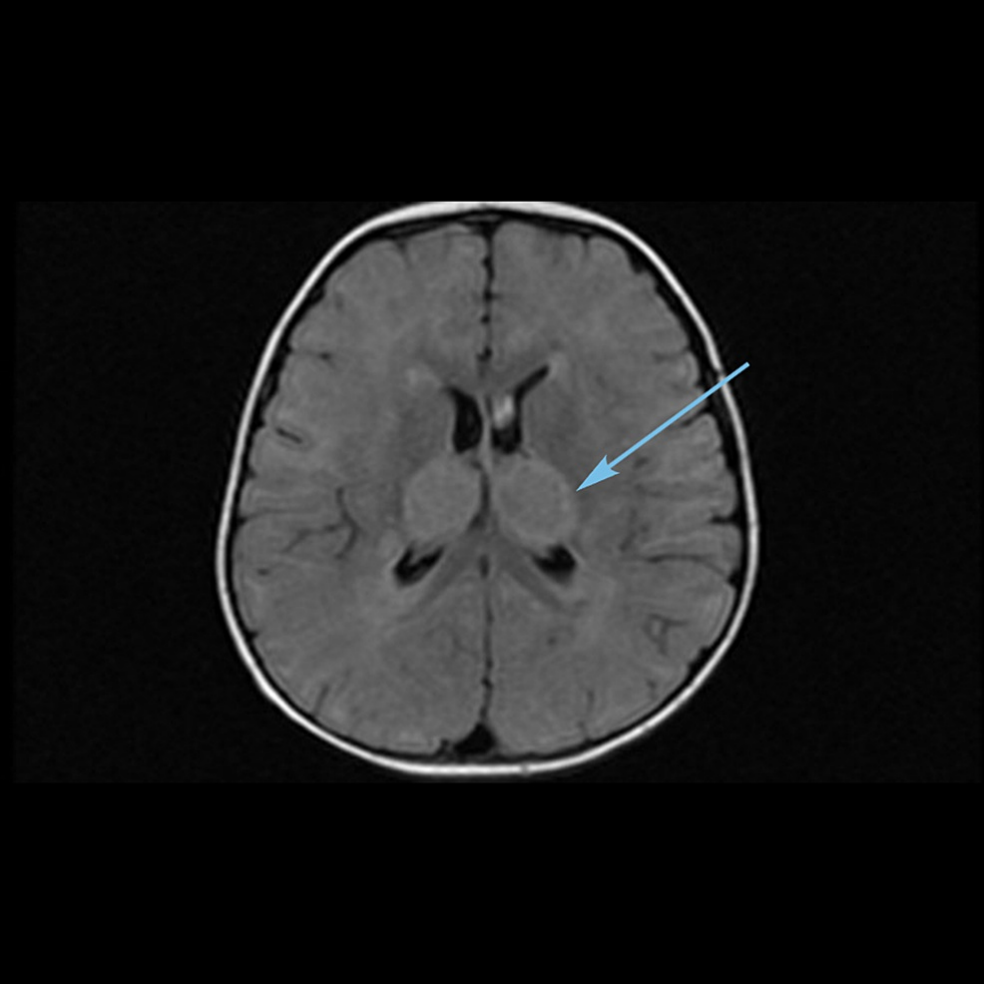
Repeat MRI brain axial image showing hemorrhagic changes in bilateral thalami (arrow).

A one-year-old, previously healthy female child, presented to the emergency room (ER) with a history of one episode of generalized tonic-clonic seizure in the setting of three-day febrile illness and one episode of loose stools. On initial assessment, she appeared irritable and mildly dehydrated with tachycardia, but she was normotensive. Her initial neurological examination was unremarkable. However, in the ER, she developed multiple seizures requiring abortive seizures medications. The initial workup showed anemia, leukopenia, and thrombocytopenia. The cerebrospinal fluid was clear with no cells, glucose level was 58 mg/dL (blood glucose level was 82 mg/dL), and protein level was 80 mg/dL, with negative gram stain and culture. In addition, she had undergone an emergency plain computerized tomography (CT) scan of the brain, which showed hypodensities in bilateral frontal white matter, thalami, and dentate nuclei. Hence, empirical antibiotics and acyclovir were immediately initiated. After her admission,* *she continued to have seizures with a further increase in irritability that became associated with dystonic posturing. Magnetic resonance imaging (MRI) of the brain (Figures 4-6) was obtained, which showed areas of diffusion restriction and hyperintensities in the bilateral dentate nuclei, thalami, and centrum semiovale in fluid-attenuated inversion recovery (FLAIR) sequences, considering acute disseminated encephalomyelitis (ADEM). So IV methylprednisone was initiated while awaiting her infectious workup results. However, a repeat MRI (Figure 7) showed new lesions with hemorrhagic changes in the thalami, suggesting a possibility of hemorrhagic ADEM. Meanwhile, her infectious workup showed a picture consistent with human herpesvirus 6 infection: HHV-6 CSF PCR: 2,750 DNA copies, HHV-6 plasma PCR: 1,630 DNA copies, HHV-6 immunoglobulin G (IgG): 1:640, and a negative HHV-6 IgM: <1:20. Therefore, cidofovir and intravenous immunoglobulin infusions were started. Her seizures were well controlled, and she was clinically better. However, she had a regression of her developmental milestones along with intermittent dystonic posturing as a sequela of HHV-6 encephalitis. In view of her presentation, an immunological workup was obtained, which revealed an underlying immunodeficiency syndrome.

## Discussion

In this case series, we illustrate the varied spectrum of clinical and radiological features of HHV-6 encephalitis in children. Clinically, acute infections of HHV-6 typically affect children younger than three years and can manifest as a benign illness characterized by fever and rash (“sixth disease” or exanthema subitum)” [1]. Despite the benign presentation of HHV-6 infection, it can cause meningitis, encephalitis, febrile seizures, and epilepsy [3]. HHV-6-related encephalitis has been commonly described as a disease of immunocompromised patients, but it still can manifest in immunocompetent patients [3]. Historically, HHV-6 viruses are less implicated and seldom evaluated in immunocompetent children with encephalitis.

HHV-6 encephalitis has various types of clinical courses, including acute necrotizing encephalopathy (ANE), hemorrhagic shock and encephalopathy syndrome (HSES), and acute encephalopathy with biphasic seizures and late reduced diffusion (AESD) [7,8]. Our case series also showed the spectrum of mild to severe patterns of encephalitis with mild meningeal enhancement in case one to acute necrotizing encephalopathy and poor neurological outcome in case three.

Autoimmune encephalitis has recently been reported following HHV-6 infection. The proposed mechanism in these diseases is induced auto-reactivity caused by antigenic exposure to HHV-6. Anti-γ-aminobutyric acid type A (GABA_A_) receptor encephalitis following HHV-6 encephalitis has been reported in an infant [9]. Anti-N-methyl-D-aspartate receptor encephalitis has also been reported in the setting of both CNS and serum HHV-6 viremia. Although the pathogenesis of HHV-6 encephalitis/encephalopathy remains unclear, it is thought that two different mechanisms, such as direct invasion (primary encephalitis) and immune-mediated impairment (secondary encephalitis), might play an important role in causing CNS manifestations [10]. 

A variety of tests are used to provide evidence of HHV-6 infection. These include isolation of either HHV-6 DNA from clinical specimens (e.g., whole blood, plasma, serum, cerebrospinal fluid, brain, lung tissue, respiratory secretions, or specimens obtained with bronchoalveolar lavage) or demonstration of a rise in HHV-6 immunoglobulin G (IgG) antibodies [11,12]. For patients experiencing primary infection, serologic studies show the appearance of specific IgM antibodies during the first week and their disappearance after one month. As for IgG antibodies, they are detected at a later period than IgM but persist indefinitely. Our facility does cerebrospinal fluid BioFire (PCR test) in all children with a suspected case of central nervous system infections, which includes PCR test for HHV-6 as well. Based on the sensitivity and specificity of current PCR techniques, the detection of HHV-6 DNA in cerebrospinal fluid (CSF) is considered sufficient for the diagnosis of an active infection of the CNS, regardless of the level of viremia [13,14].

Most patients with HHV-6 encephalitis have signal intensity abnormalities in the hippocampal formation and amygdala. Some also have the involvement of limbic structures outside of the medial temporal lobe [15]. Human herpesvirus 6 encephalitis has been associated with a myriad of neuroradiographic findings like striatal necrosis and white matter abnormalities [16].

Not only is this underdiagnosed, but no controlled trials have evaluated the clinical efficacy of antiviral agents or validated treatment indications of HHV-6 encephalitis. Plausible treatment strategies based on case reports include ganciclovir, foscarnet, cidofovir, and brincidofovir [17]. Because reports of HHV-6 resistance to ganciclovir have been increasing, foscarnet may be the treatment of choice for patients with HHV-6 encephalitis [18]. Moreover, foscarnet is currently considered the preferential treatment option for HHV-6 encephalitis in patients with anemia, as the administration of ganciclovir poses an additional risk of dose-limiting hematological toxicity [19,20], which happened to our first case and was a reason to hold it.

The prognosis of HHV-6 infection is usually excellent. However, deaths and various neurological deficits have been reported in encephalitis complicated illness [1]. In our cases, patients showed marked clinical responses to antiviral therapy. Two of our patients had returned back to normal baseline, and one patient had persistent neurological deficits as a complication of the infection. This adds and supports several case reports and case series in the literature that have suggested improvement in presumed HHV-6 encephalitis after administration of foscarnet or ganciclovir. 

## Conclusions

It is important to consider HHV-6 infection as a diagnostic possibility in any children with acute central nervous system infection with white matter lesions. Systematic studies are needed to formulate a universal diagnostic algorithm and treatment protocol for this poorly understood common childhood viral infection.
